# Association between Malnutrition and Quality of Life in Elderly Patients with Rheumatoid Arthritis

**DOI:** 10.3390/nu13041259

**Published:** 2021-04-12

**Authors:** Wojciech Tański, Justyna Wójciga, Beata Jankowska-Polańska

**Affiliations:** 1 Department of Internal Medicine, 4th Military Teaching Hospital, 50-981 Wrocław, Poland; wtanski@op.pl; 2 Student Research Club at the Division of Nursing in Internal Medicine, Department of Clinical Nursing, Wrocław Medical University, 51-618 Wrocław, Poland; badanianaukowe@4wsk.pl; 3 Department of Clinical Nursing, Wroclaw Medical University, 51-618 Wrocław, Poland

**Keywords:** malnutrition, quality of life, rheumatoid arthritis, frailty syndrome

## Abstract

Rheumatoid arthritis (RA) is a progressive articular disease. In addition to damaging the joints, it may cause multiple organ complications, and considerably impair the patient’s functioning. Elderly patients with RA report pain, fatigue, mood disorders, sleep disorders and insomnia, accompanied by weakness, poor appetite, and weight loss. All these factors combined have an adverse effect on the patient’s perceived quality of life (QoL). Due to the chronic nature of RA and the high risk of malnutrition in this patient group, the present study investigated QoL, activities of daily living, and frailty syndrome severity in relation to MNA (Mini Nutritional Assessment) questionnaire scores among elderly RA patients. The study included 98 patients (aged over 60) diagnosed with RA per the ARA (American Rheumatism Association) criteria. The following standardized instruments were used: WHOQoL-BREF for QoL, the Edmonton Frail Scale for frailty syndrome severity, MNA for nutritional status assessment, and MMSE (Mini-Mental State Examination) to assess any cognitive impairment. Medical data were obtained from hospital records. Patients with a different nutritional status differed significantly in terms of limitations in activities of daily living (ADL) and instrumental activities of daily living (IADL). Higher levels of malnutrition were associated with greater limitations in activity. An adverse impact of lower body weight on cognitive function was also observed (dementia was identified in 33.33% of malnourished patients vs. 1.79% in patients with a normal body weight). Likewise, frailty was more common in malnourished patients (mild frailty syndrome in 33.3%, moderate in 16.67%, and severe in 16.67%). Malnourished patients had significantly lower QoL scores in all WHOQoL-BREF questionnaire domains than those with a normal body weight, and multiple-factor analysis for the impact of selected variables on QoL in each domain demonstrated that frailty was a significant independent determinant of poorer QoL in all domains: perceived quality of life (β = −0.069), perceived health (β = −0.172), physical domain (β = −0.425), psychological domain (β = −0.432), social domain (β = −0.415), environmental domain (β = −0.317). Malnutrition was a significant independent determinant of QoL in the “perceived health” domain (β = −0.08). In addition, regression analysis demonstrated the positive impact of male sex on QoL scores in the psychological (β = 1.414) and environmental domains (β = 1.123). Malnourished patients have a lower QoL than those with a normal body weight. Malnutrition adversely affects daily functioning, cognitive function, and the severity of frailty syndrome. Frailty syndrome is a significant independent determinant of poorer QoL in all WHOQoL BREF domains.

## 1. Background

Rheumatoid arthritis (RA) is a chronic polyarticular inflammatory disease affecting 1% of the total population [1]. In Poland, approximately 400,000 people suffer from RA [2]. Approximately 30% of RA cases are patients over 60 years of age, which is particularly important in view of the increasing survival time, as it means that the number of elderly RA patients will continue to grow [3]. Rheumatoid arthritis is a progressive articular disease. As well as damaging the joints, it may cause multiple organ complications, and restrict a patient’s physical, psychological, and social functioning to a considerable extent. Elderly patients with RA may be treated less aggressively than they should [4,5] and are less likely to receive combination therapy with DMARDs (disease-modifying antirheumatic drug) or biologics compared to younger patients. Treatment may have different purposes: for an old person it may help maintain function and independence now and in the short term, whereas for a young person it is to help now and in the long-term future (to prevent damage). The available studies demonstrate an adverse impact of the disease on patients’ daily functioning, as the patients report very poor health outcomes in all aspects of daily living [6,7,8]. The disease leads to dissatisfaction and distress associated with the physical, professional, psychological, and social limitations it imposes. Besides difficulties in daily activities, patients complain of pain, fatigue, mood disorders, and insomnia. The disease tends to be very distressing, as it interferes with the performance of social roles, and causes pain. In addition to the typical bone and joint mobility restrictions and joint pain, RA produces systemic symptoms. The most commonly reported ones include loss of appetite, body weight loss, weakness, fatigue, and sleep disorders. RA has also been linked to multiple gastrointestinal complaints, specifically including dyspepsia (upper abdominal pain, burning, postprandial fullness and early satiety, bloating, nausea, and belching), mucosal ulcers, and changes in bowel habits, with constipation or diarrhea [9]. Among patients with severe RA, nutritional deficiency concerns are diagnosed and most often concern: folic acid, vitamin D, and zinc deficiency, retinol-binding pro-tein (RBP) and thyroxine-binding prealbumin (TBPA). Nutritional parameters are related to disease activity and glucocorticoid treatment. Prolonged use of these may cause intestinal problems such as irritation, ulcers, acid reflux and even kidney failure. Medicaments used to control the activity of the disease causes gastrointestinal changes, which affect the ingestion, digestion and absorption of food [10]. Thirty two percent of patients with RA experience rheumatoid cachexia, as a result of joint destruction, subsequent muscle inactivity, high levels of sarcoactive inflammatory cytokines—incl tumor necrosis factor α (TNF) -α and interleukin (IL) 1β, loss of muscle mass and strength and the accompanying increase in fat mass are very common in patients with rheumatoid arthritis. In addition, factors used commonly in RA to tamper down inflammation, including TNF inhibitors and the IL 6 receptor blocker, further aggravate body protein and energy metabolism, inducing body composition alterations [11].

Elderly patients with RA next to malnutrition pain, report fatigue, mood disorders, sleep disorders and insomnia, accompanied by weakness, poor appetite, and weight loss. When combined, all these factors adversely affect the patient’s perceived quality of life [12,13]. The prevalence of malnutrition in chronically ill patients and its adverse impact on chronic treatment outcomes, morbidity, and QoL have been well documented, though studies specifically focusing on rheumatic disease remain scarce [14,15]. Patients with RA have a characteristic pattern of malnutrition, with mild obesity at the early stage of the disease [16]. As the condition progresses, skeletal muscle protein levels drop and BMI (Body Mass Index) continually decreases, mainly due to rheumatoid cachexia, loss of lean body mass, and metabolic disturbances caused by increased proinflammatory cytokine levels. Such a BMI decrease and malnutrition in RA patients is a predictor of poor prognosis in terms of functioning and life expectancy [16]. Patients also have concurrent mental health problems, such as depressive disorders or chronic fatigue, which may additionally suppress appetite and limit food intake [17].

Due to the long-term chronic disease processes, many patients with advanced RA are elderly, which is associated with a risk of comorbidities typical for the elderly population. The most common ones include cognitive impairment, frailty syndrome, polypharmacy, and multimorbidity. The adverse impact of malnutrition on morbidity, mortality and QoL in a variety of diseases has already been recognized [18,19,20]. The available publications report associations between the above-mentioned disorders typical for the elderly population and malnutrition in patients with cardiovascular disease [18], respiratory disorders [19], or cancers [20], but few papers have been published on patients with rheumatic diseases. Therefore, we have undertaken to evaluate nutritional status, cognitive impairment, functional limitations, frailty syndrome severity, and their associations with QoL.

QoL evaluation has become an important part of daily practice for physicians and nurses in recent years, and a topic of particular interest for many researchers. It may constitute an important endpoint in the evaluation of treatment outcomes. Approaching treatment effectiveness from the point of view of QoL has presented the medical community with an opportunity to demonstrate long-term effectiveness in the management of chronic diseases.

The ongoing assessment of QoL is a multi-dimensional concept incorporating the individual’s perception of health status, psycho-social status and other aspects of life and rather concerns the impact of disease and symptoms on daily functioning and perception of health. Quality of life is assessed on the basis of physical, mental, social and environmental fitness, which refers to mobility, financial resources, access to medical care and the home environment. Additionally, questions are asked about the perception of health and quality of life. The purpose of our study was to analyze selected factors, including cognitive impairment, frailty syndrome, multimorbidity, the number of medications taken, and patient sex, in the context of malnutrition, and to evaluate their relationships with QoL in elderly RA patients.

## 2. Material and Methods

### 2.1. Study Course

The study included 98 patients hospitalized at the Department of Rheumatology and Internal Diseases. The inclusion criteria were: age above 60 years, stable clinical condition and RA diagnosed per the ARA criteria [21]. Patients with severe cognitive impairment preventing full contact (MMSE, score below 23) were excluded from the study. Patients were assessed with regard to the inclusion criteria during their visit at the department. The inclusion procedure was performed by properly trained staff (a physician and a nurse).

The study plan had assumed that patients would complete the surveys without assistance, but in some cases, due to the advanced age and vision impairment of some included patients, a nurse assisted the respondents in reading the questionnaire items whenever necessary. In all cases, the primary consideration was the unassisted provision of responses to all items.

All elderly patients included in the study were evaluated on the basis of an interview questionnaire specifically designed for the purpose of the study, and a number of standardized scales. The MMSE questionnaire was used to evaluate cognitive function for the purpose of patient exclusion based on the pre-established criteria.

**Figure 1:**
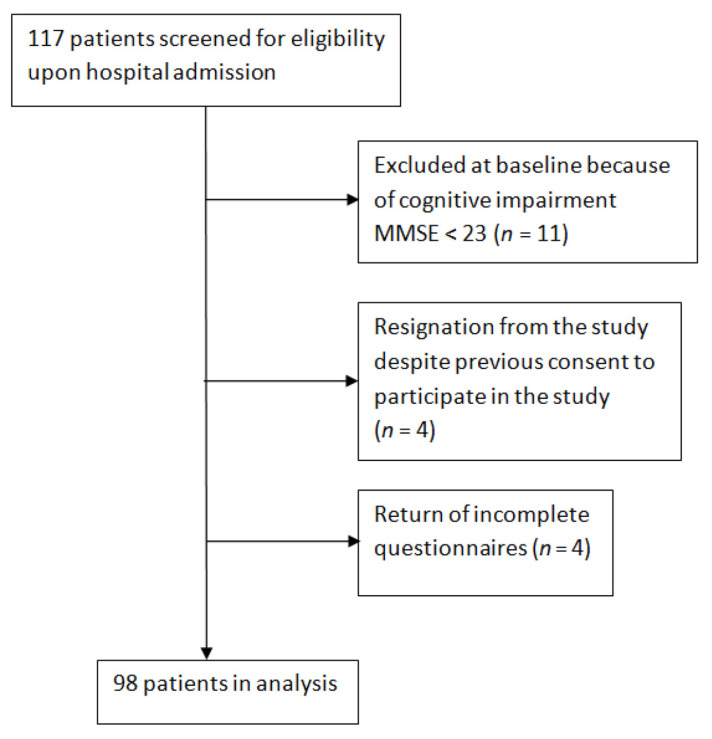
Flowchart of the study population.

The first stage of the study involved an interview used to collect general personal details and data on respondents’ socio-economic standing and health, and the MMSE assessment (Figure 1). During the study period, 117 patients matching the age and RA diagnosis criteria were enrolled in the study, but 11 were then excluded based on MMSE scores. Further evaluations included the 106 patients who met the inclusion criteria.

At the next stage of the study, 106 patients were evaluated for functional capacity, nutritional status, and QoL. At this stage, 4 patients dropped out, despite their prior consent to participate in the study, and another 4 patients returned incomplete questionnaires.

All the remaining 98 patients were in a stable clinical condition. All patients consented to participate, having been informed that this was strictly voluntary and anonymous, and that they could withdraw at any time without providing a reason.

The study was approved by the Bioethics Committee of Wrocław Medical University.

### 2.2. Data Collection

The authors’ own questionnaire was used to collect data on patients’ sex, age, education, relationship status and residence. Clinical data were gathered from hospital records.

### 2.3. Cognitive Function Assessment

We used the Folstein Mini-mental State Examination (MMSE) questionnaire. It is commonly used for dementia screening, allowing for rapid administration and straightforward interpretation of results [22]. The MMSE measures cognitive functions including sense of direction, memory, attention, linguistic function, and visual–spatial abilities, as well as the ability to count, recall things, repeat, and carry out orders [22]. The score range is 0–30, and lower scores indicate poorer cognitive function. Scores ≤ 23 suggest cognitive impairment. MMSE Cronbach alpha was 0.6108 [23].

### 2.4. Functional Capacity Assessment

The Instrumental Activities of Daily Living (IADL) scale was used to assess patients’ ability to perform complex daily activities. The scale evaluates eight parameters: using a phone, shopping, preparing meals, housekeeping, doing laundry, using transportation, taking medication, and managing money. Scores for each category range from 3 points—the patient is able to perform the activity with no assistance, to 1 point—the patient is entirely unable to perform the activity. The maximum score is 24 points, and the lower the score, the less independent the respondent [24]. The Lawton IADL subscales had a Cronbach’s alpha of 0.91 [25]. Cronbach’s alpha for the Polish study group was 0.93 (0.91; 0.95).

### 2.5. WHOQoL-BREF Quality of Life Assessment

The standardized, short version of the questionnaire is used for subjective QoL assessment in healthy or ill individuals, for research or clinical purposes. It provides a QoL profile including four domains: physical, psychological, social, and environmental. Scores in each domain are determined by calculating arithmetical means from all items contained in a given domain (26 items total). Domain scores reflect patients’ individual perceptions of their QoL in each aspect. The Cronbach’s alpha coefficient for the original version of the WHOQOL-BREF scale was 0.896, and for the Polish version in the own study, 0.84 (0.79; 0.88) [26,27].

### 2.6. The Mini Nutritional Assessment (MNA) Questionnaire

All patients were tested using the complete MNA^®^ Mini Nutritional Assessment questionnaire (Nestlé Nutrition Institute), comprising a screening part and an assessment part. The former concerns appetite loss, mobility, weight loss over 3 months, BMI, and neuropsychological problems. The latter concerns diet (number of meals, food and fluid intake), medication, self-reported nutritional status, and overall health. Calf and mid-arm circumference measurement is also included. The instrument has strict cut-off values, which has contributed to its widespread use in clinical practice worldwide. The assessment part comprises 12 items, broken down into anthropometric, general, dietary, and subjective assessment. Total MNA scores from both parts are used to identify patients with a normal nutritional status (≥24 points), at risk of malnutrition (17–23 points), and with protein-calorie malnutrition (<17 points) [28]. MNA scale has good internal consistency. The Cronbach’s Alpha, were 0.83. Validation studies have shown high reliability and validity of this tool (scale sensitivity—97.9%, scale specificity—100%) [29].

### 2.7. Statistical Analysis

For all patients included, we analyzed the correlations between nutritional status assessed using the Mini Nutritional Assessment scale and cognitive function, frailty, and WHOQoL-BREF scores. We also considered differences resulting from socio-demographic parameters, health status, and clinical parameters (number of chronic conditions). MNA scores below 17 points were used to identify malnutrition.

For continuous or ordinal variables, data are presented as means and standard deviations, and as medians with interquartile ranges and ranges; for categorical variables, data are presented as percentages. Comparisons of categorical variable frequencies between groups were performed using the chi-squared test or Fisher’s exact test (when class size was smaller than five). Quantitative variable values were compared between two groups using the Mann–Whitney test. For three or more groups, quantitative variable values were compared with the use one-way ANOVA or the Kruskal–Wallis test (when the variable did not have normal distribution). The effect of the MNA group on WHOQOL-BREF scores was analyzed with the use of ANCOVA with the age as covariate of WHOQOL-BREF. When a comparison showed statistically significant differences, post-hoc analyses were performed using the Bonferroni or Dunn’s or test, respectively. In the case of heavily left-skewed IADL scores, the effect of age was verified in a two-stage approach. Firstly, a linear regression model of the relationships between the age and IADL score was performed and the residuals were derived. At the second stage, the effect of MNA groups on the residuals (logarithmized for normalizing its frequency distribution) was tested with the use of one-way ANOVA. The relationships between selected variables and WHOQOL-BREF was analyzed with the aid of full multiple linear regression model. The Variance Inflation Factor (VIF) was used for checking the co-linearity of these explaining variables. The VIF ranged from 1.05 (Sex) do 2.38 (EFS), which proves the weak co-linearity of the variables. All analyses used a significance threshold of 0.05. The analyses were performed using the R software, version 4.0.4 (multiple regressions and the VIF calculation), the G*Power software (power test analyses), and Statistica 13.5 (other analyses).

## 3. Results

The MNA scores in our study were interpreted in accordance with the key and 57.14% patients were found to have a normal nutritional status, 36.73% were at risk of malnutrition, and 6.12% were malnourished. Further analyses were performed for groups identified on the basis of patients’ nutritional status.

A comparative analysis of the socio-demographic characteristics of the patients studied and their nutritional status demonstrated that malnourished patients were significantly more likely to suffer from colorectal disease (50% vs. 13.9% vs. 11.8%; *p* = 0.001) compared to those RA patients who were well-nourished or at risk of malnutrition (Table 1). Notably, the malnourished patients were also significantly older than the other two groups (73.7 (7.4) vs. 72.5 (6) vs. 71.1 (6.8), *p* = 0.03). In the Bonferroni test, significant differences were observed between the Malnutrition group and the Normal nutritional status group *p* = 0.045).

### 3.1. ADL and MNA

Patients with a different nutritional status differed significantly in terms of impairment in activities of daily living. Table 2 shows significant statistical differences in the share of ADL groups in the groups depending on the nutritional status. In the Poorer nutritional status group, the most people who were unable to function independently were observed (50 vs. 13.9 vs. 0.0); 98.2% (*p* < 0.001) of normally nourished patients had a full ADL capacity, while in the malnourished group, one in two patients, had a severely limited capacity to perform ADL.

### 3.2. IADL and MNA

IADL scores were heavily left-skewed in the groups analyzed and therefore, the Kruskal–Wallis test was used, and results were presented as medians, quartiles, and ranges.

**Figure 2:**
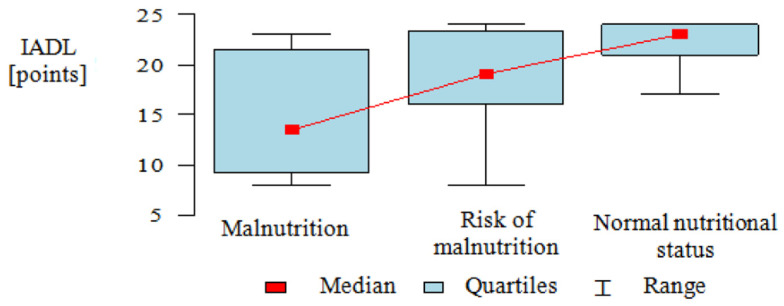
Ability to perform instrumental activities of daily living by nutritional status.

Patients with a different nutritional status differed significantly in terms of limitations in instrumental activities of daily living. To accurately identify correlations between the two factors, post-hoc analysis was performed. It demonstrated that normally nourished patients had significantly higher IADL scores than patients at risk of malnutrition and malnourished patients, while the difference between malnourished patients and patients at risk of malnutrition was statistically insignificant (Dunn’s test: *p* < 0.001, *p* = 0.006, *p* = 0.73, respectively) (Table 3, Figure 2).

### 3.3. Edmonton Frail Scale (EFS) and MNA

Furthermore, the relationship between nutritional status and frailty syndrome was evaluated in the study sample. The results demonstrated an association between greater degree of malnutrition and greater severity of frailty in RA patients. Among the malnourished patients, 16.67% had severe frailty and the same percentage had moderate frailty, while in the normally nourished group, there was no severe frailty, and moderate frailty was only found in 3.57% of patients (*p* < 0.001). In the malnourished group, all patients had some degree of frailty, while nearly half of patients with a normal nutritional status had no frailty.

### 3.4. MMSE and MNA

Our findings show that patients with a different nutritional status also differed significantly in terms of cognitive impairment, as more impairment was associated with poorer nutrition (Table 4). Only half of the malnourished respondents had a normal cognitive function test result, while in the normally nourished group, the percentage was 87.5% (*p* = 0.008).

### 3.5. Comparison of QoL in Specific WHOQoL Domains by Nutritional Status

WHOQoL-BREF scores did not have normal distributions in the groups analyzed (Shapiro–Wilk test *p* < 0.05), and therefore, the Kruskal–Wallis test was used for analysis, and the results were presented as medians, quartiles, and ranges for each variable.

Our findings demonstrated an association between QoL in the social and physical domains and nutritional status. To accurately identify correlations between the two factors, post-hoc analysis was performed. It showed that in each of the two domains, patients at risk of malnutrition had a poorer QoL than those with a normal nutritional status (Table 5). The mean score in the physical domain was 10.83 in the malnourished group and 12.68 in the normally nourished group. Likewise, the mean score in the social domain was 12.33 for malnourished patients, and 14.29 for those with a normal nutritional status. Similar relationships were observed for the perceived QoL and perceived health domains, where patients with a normal body weight scored higher than those who were at risk of malnutrition or malnourished: 3.68 ± 0.58 vs. 3.33 ± 0.68 vs. 3.33 ± 1.03, respectively, for perceived QoL, and 3.14 ± 0.98 vs. 2.47 ± 0.84 vs. 2.67 ± 0.82 for perceived health. The age effect on the WHOQOL-BREF score was poor (significant in environmental domain only). The results are shown in Table 5.

### 3.6. Multiple-Factor Analysis for the Impact of Selected Variables on WHOQoL-BREF Domain Scores

We analyzed the impact of selected variables (nutritional status, cognitive function, frailty, chronic comorbidities, sex, and number of medications taken) on each WHOQOL-BREF domain. Linear regression model findings are as shown in Table 6.

For the “perceived QoL” domain, frailty was found to be an independent predictor of QoL. Each additional point in the EFS questionnaire decreased the QoL score in this domain by an average of −0.07 points (*p* < 0.05). For the “perceived health” domain, independent predictors included frailty and cognitive impairment. Each additional point in the EFS questionnaire decreased the QoL score in this domain by an average of −0.17 points (*p* < 0.001). For the physical QoL domain, frailty was an independent predictor. Each additional point in the EFS questionnaire decreased the QoL score in this domain by an average of −0.50 points (*p* < 0.001). As to medication (>3), it increased QoL in the domain by an average of 1.71 (*p* < 0.05). For the psychological QoL domain, independent predictors included frailty and male sex. Each additional point in the EFS questionnaire decreased the QoL score in this domain by an average of −0.36 points (*p* < 0.01). As to the male sex, it increased QoL in the domain by an average of 1.38 (*p* < 0.05), compared to female sex. For the social QoL domain, frailty was an independent predictor. Each additional point in the EFS questionnaire decreased the QoL score in this domain by an average of −0.34 points (*p* < 0.001). For the environmental QoL domain, independent predictors included frailty and male sex. Each additional point in the EFS questionnaire decreased the QoL score in this domain by an average of −0.61 points (*p* < 0.01), while male sex increased it by an average of 1.93 points (*p* < 0.05), compared to female sex. As to age, it increased QoL in the domain by an average of 0.18 (*p* < 0.01).

## 4. Discussion

Our study demonstrated that among RA patients, health-related QoL as measured using the standardized WHOQoL-BREF scale was poorer in malnourished than in properly nourished patients. In addition, we found that poor nutritional status was associated with cognitive impairment and frailty syndrome severity, all of which adversely affected daily functioning and may in turn result in poorer nutrition. It is worth noting that in the regression analysis, the nutritional status was not a statistically significant factor affecting QOL, different to what we assumed in our study.

Musculoskeletal disorders can result in a loss of the ability to work and lower QoL [2]. Though effective pharmaceutical treatments are available, the continued high burden of comorbidities and impaired functional capacity indicate that further innovation in RA management is required. Diet and compensation of lost body weight have proven to be promising strategies for reducing the burden of RA [9].

Our study is one of only a few on the topic. Notably, hospitals have recently been obliged to assess patients’ nutritional status, but the results of these assessments are not widely used to evaluate patients’ functioning and treatment outcomes. The authors emphasize that malnutrition may result from improper nutrition [30].

Malnutrition has been demonstrated to affect QoL chronically ill elderly populations [31], but issues of malnutrition in rheumatology are rarely addressed in studies. To date, only a few papers have been published on the relationship between BMI and QoL in this patient group. In Fu-kuda et al.’s study, QoL was poorer in RA patients with a low BMI than in those with a moderate BMI [32]. The loss of muscle protein, but not of adipose tissue, is a major factor in QoL deterioration, regardless of disease activity. Therefore, according to Fu-kuda et al., RA management should also include nutritional treatment and the prevention of further muscle protein loss, which may significantly improve QoL [32].

Kremers et al. demonstrated that joint pain and fatigue in severe forms of RA were not the main factors contributing to QoL deterioration in RA patients with a low BMI [33]. Severe, chronic inflammation can be an aggravating factor, but the authors reported that comorbidities, including cardiovascular disease, could have been responsible for the poor QoL in RA patients with a low BMI [33]. The literature proves the relationship between the occurrence of comorbidities and the quality of life. In our study, despite the fact that comparative analyses showed differences in the group of patients diagnosed with Colorectal diseases, the regression analysis did not show co-existence of chronic disease as a variable related to QoL. The quality of life of the sick is the result of a single and unique interaction of the influence of the disease, the patient’s individual abilities, adaptation forces and coping processes of dealing with the disease [34]. In our study, another determinant related to the assessment of the quality of life, but only in the perceived health domain, was cognitive function. A higher rating of cognitive functions lowered the quality of life rating in this domain. Cognitive disorders are associated with information processing; as they slow down, performing demanding tasks is difficult and requires more effort. There is a “stiffness” of thinking and a susceptibility to distractors and perseveration. Subjective health assessment is based on a personal assessment of the ailments and symptoms constituting sequelae of the disease. People with cognitive functions disorder may have a disturbed perception of reality and an objective insight into their health condition, treatment consequences and long-term functioning possibilities, thus overstating their assessment.

In our findings, the adverse impact of frailty syndrome on QoL was observed. Malnutrition-related loss of muscle protein leads to physical disability and limitations in ADL and IADL, which are characteristic of people with frailty syndrome [32]. Tada et al. identified frailty syndrome in 18.9–38.9% of RA patients, and its prevalence was significantly higher in those with sarcopenia and poor disease control [35]. The specific components of frailty syndrome are also worth recognizing, as its pathogenesis is often linked to malnutrition and lack of physical activity.

Marcora et al. claim that the implementation of rehabilitation and nutritional programs with protein supplementation may play a significant role in improving QoL in a frailty patient group. This may certainly also limit the adverse consequences of frailty [36]. Marcora et al. report that a diet preventing the accumulation of excess lipids and the loss of muscle protein should have a high protein and low-fat content, with a controlled supply of carbohydrates. According to these authors, such a diet may lead to improved QoL in RA patients by preventing the loss of muscle protein and maintaining BMI in the normal range [37].

In our study, frailty was the strongest negative independent determinant of QoL in all the QoL domains studied. Salaffi et al., in a study on frailty in RA patients, demonstrated that this patient population was at very high risk of developing frailty syndrome. According to the authors, this may be related to the functional deterioration, depression, cognitive impairment, falls, malnutrition and polyphagia they ex-perience [38].

Moreover, in our own study, we proved clearly that a greater degree of malnutrition is associated with a greater severity of frailty syndrome and cognitive impairment. Notably, concurrent frailty syndrome complicates patient management due to multi-morbidity and risk of progression all the way to irreversible disability [39]. Bąk et al. demonstrated a correlation between frailty syndrome and DAS28 (Disease Activity Score) in a group of RA patients, which means that frail patients have a more advanced disease, more severe pain, and a higher degree of disability [40]. Furthermore, in a Bąk et al. study as in our own study, frailty was not an independent determinant of quality of life but negatively affected every day functioning. They also report that QoL is reduced by disease symptoms, which are more severe in those RA patients who have concurrent frailty.

Another independent determinant that had a positive impact on the QoL assessment in the psychological domain was male gender. The issue of gender as a determinant of QoL is often discussed and researchers lack a unified position. The available studies indicate a lower quality of life of the respondents, which may result from a negative attitude to pain and the occurrence of deformities. In women, the disease has a negative impact on social life.

It is worth emphasizing that the quality of life depends on adaptive forces and the processes of coping with the disease, which may be justified by the results obtained by us.

Implications for Practice: The issues addressed here, concerning the prevalence and importance of frailty and malnutrition in RA patients, represent a novel and under-researched topic in rheumatology. Therefore, further studies are warranted to understand whether improved nutritional status would be associated with improved cognitive function, reduced frailty, and better QoL. RA patients should be routinely screened for malnutrition and frailty, so that preventive measures can be implemented to reduce the risk of these conditions and minimize their QoL impact.

Study Limitations: Our study is not free of limitations. The relatively small study sample may be considered one such limitation; it did, however, include a specific group of individuals aged 60 and above, diagnosed with rheumatoid arthritis. Moreover, frailty syndrome was only evaluated using a single self-reported instrument, though no consensus has yet been reached regarding the instrument to use in such evaluations. In addition, we only used a generic QoL questionnaire.

## 5. Conclusions

In conclusion, malnourished RA patients had a significantly impaired QoL—more malnutrition was associated with poorer QoL. QoL included perceived health, social and physical domains. It was not, however, an independent determinant of lower QoL. Malnutrition was significantly associated with cognitive impairment and with frailty syndrome severity. Frailty was a strong and independent predictor of poorer QoL. Male sex and age was associated with better QoL in the environmental domain and Medication >3 was an independent predictor of better QoL in the physical domain.

## Figures and Tables

**Table 1:** Socio-demographic and clinical characteristics in the studied groups, by nutritional status (Fisher test).

Characteristic		Malnutrition (*n* = 6)	Risk of Malnutrition (*n* = 36)	Normal Nutritional Status (*n* = 56)	All (*n* = 98)	*p* *
		%				
Sex	Female	50.0	61.1	69.6	65.3	0.45
	Male	50.0	38. 9	30.34	34.7	
Relationship status	In a relationship	50.0	41.7	64.3	55.1	0.29
	Single	50.0	58.3	35.7	44.1	
Education	Primary	16.7	8.33	7.1	8.2	0.60
	High school or vocational	83.3	61.1	60.7	62.2	
	College/university	0.0	22.2	28.6	24.5	
	No data	0.0	8.3	3.6	5.1	
Financial standing	Very good	0.0	0.0	3.6	2.0	0.83
	Good	50.0	33.3	41.1	38.8	
	Moderate	50.0	61.1	51.8	55.1	
	Poor	0.0	5.6	3.6	4.18	
Comorbidities	Colorectal diseases	50.0	13.9	11.8	9.2	0.001
	Asthma	0.0	11.1	14.3	12.24	0.89
	Hypertension	33.3	63.9	53.6	56.12	0.29
	Diabetes mellitus	0.00	30.56	26.79	26.53	0.35
	Thyroid disease	16.67	27.78	8.93	16.33	0.05
	Renal insufficiency	0.00	11.11%	0.00	4.08	0.05
Age [years]	mean ± SD	73.7 ± 7.4	72.5 ± 6	71.1 ± 6.8	72.6 ± 6.5	0.03

*n*: number; SD: standard deviation; *: significance level.

**Table 2:** Independence in daily activities by nutritional status.

	Malnutrition (*n* = 6)	Risk of Malnutrition (*n* = 36)	Normal Nutritional Status (*n* = 56)	All (*n* = 98)	*p* *
	%				
ADL—activities of daily living—unable to function independently	50.0	13.9	0.0	8.2	<0.001
ADL—activities of daily living—some restrictions	0.0	11.1	1.8	5.1	
ADL—activities of daily living—unrestricted independent functioning	50.0	75.0	98.2	86.7	

* Fisher’s exact test; ADL: activities of daily living; *n*: number.

**Table 3:** Ability to perform instrumental activities of daily living by nutritional status.

	IADL (Points)						*p* *
	*n*	Mean	SD	Median	Min.	Max.	
Malnourished	6	15	6.96	13.5	8	23	
At risk of malnutrition	36	19.11	4.46	19	8	24	
Normal nutritional status	56	22.54	1.8	23	17	24	
All	98	20.82	4.05	22	8	24	<0.001

* Kruskal–Wallis test + post-hoc analysis (Dunn test); *n*: number; SD: standard deviation; IADL: instrumental activities of daily living.

**Table 4:** Prevalence of frailty and cognitive impairment by nutritional status.

	Malnourished	Risk of Malnutrition	Normal Nutritional Status	All	*p* *
	%				
Frailty Syndrome					
No frailty	0	13.89	73.21	46.94	<0.001
Vulnerability	33.33	30.56	12.50	20.41	
Mild frailty	33.33	27.78	10.7	18.37	
Moderate frailty	16.67	27.78	3.57	13.27	
Severe frailty	16.67	0	0	1.02	
Cognitive Impairment (MMSE)					
Dementia	33.33	19.44	1.79	10.20	0.008
Cognitive impairment without dementia	16.67	19.44	10.71	14.29	
Normal function	50.00	61.11	87.50	75.51	

* Fisher’s exact test; MMSE: Mini-Mental State Examination.

**Table 5:** Comparison of QoL in specific WHOQoL-BREF domains by nutritional status.

WHOQOL-BREF	MNA			*p*_MNA_	*p*_Age_
	Malnourished Mean ± SD	At Risk of Malnutrition Mean ± SD	Normal Nutritional Status Mean ± SD		
Perceived Quality of Life	3.33 ± 1.03	3.33 ± 0.68 ^a^	3.68 ± 0.58 ^a^	0.049	0.93
Perceived Health	2.67 ± 0.82	2.47 ± 0.84 ^a^	3.14 ± 0.98 ^a^	0.004	0.75
Physical Domain	10.83 ± 3.49	11.31 ± 2.39	12.68 ± 2.27	0.049	0.24
Psychological Domain	12.5 ± 3.89	13.28 ± 2.73	14.45 ± 2.24	0.12	0.62
Social Domain	12.33 ± 2.86	12.67 ± 2.93 ^a^	14.29 ± 2.61 ^a^	0.036	0.14
Environmental Domain	13.83 ± 2.86	13.22 ± 2.28	13.96 ± 2.17	0.054	0.02

*p*_MNA_—statistical significance of MNA effect, *p*_Age_—statistical significance of age as a covariate of WHOQOL-BREF scores in ANCOVA test, ^a^—variables with statistical significant difference in post hoc Bonferroni test; MNA: Mini Nutritional Assessment; SD: standard deviation.

**Table 6:** Multiple linear regression model (coefficients are given) for the impact of selected variables on WHOQoL-BREF domain scores.

Variable	Perceived Quality of Life		Perceived Health		Physical Domain		Psychological Domain		Social Domain		Environmental Domain	
Intercept	2.10		5.16	**	16.95	**	14.84	*	11.74	**	15.43	
Sex (male)	−0.04		−0.17		0.79		1.38	*	0.55		1.93	*
Age	0.01		0.01		0.07		0.04		−0.03		0.18	**
EFS	−0.07	*	−0.17	***	−0.50	***	−0.36	**	−0.34	***	−0.61	**
MNA	0.01		0.00		0.02		0.09		−0.02		0.03	
Medication (>3)	0.19		−0.11		1.71	*	0.93		0.05		−0.38	
MMSE	0.03		−0.07		0.02		0.03		0.07		0.02	
Comorbidities	−0.05		−0.08		0.14		0.13		0.24		0.28	
*p* (model)	0.009		<0.001		<0.001		0.002		0.001		0.002	
R2adj.	0.12		0.23		0.21		0.16		0.20		0.16	

MNA: Mini Nutritional Assessment; EFS: Edmonton Frail Scale; MMSE: Mini-Mental State Examination. Statistical significance of variables: * *p* < 0.05, ** *p* < 0.01, *** *p* < 0.001. R2adj.—adjustedR2.

## Data Availability

The data are not publicly available due to privacy and ethical restrictions. The data presented in this study may be available conditionally from the corresponding author.

## References

[1] Sokka T., Kautiainen H., Pincus T., Verstappen S.M., Aggarwal A., Alten R., Andersone D., Badsha H., Baecklund E., Belmonte M. (2010). Work disability remains a major problem in rheumatoid arthritis in the 2000s: Data from 32 countries in the QUEST-RA Study. Arthritis Res. Ther..

[2] Kwiatkowska B., Raciborski F., Maślińska M., Kłak A., Gryglewicz J., Samel-Kowalik P. Wczesna Diagnostyka Chorób reumatycznych—Ocena Obecnej Sytuacji i Rekomendacje Zmian. https://spartanska.pl/wp-content/uploads/raport_wczesna_diagnostyka_ChR.pdf.

[3] Van Schaardenburg D., Breedveld F.C. (1994). Elderly-onset rheumatoid arthritis. Semin. Arthritis Rheum..

[4] Tutuncu Z., Kavanaugh A. (2007). Rheumatic disease in the elderly: Rheumatoid arthritis. Rheum. Dis. Clin. North Am..

[5] Ruban T.N., Jacob B., Pope J.E., Keystone E.C., Bombardier C., Kuriya B. (2015). The influence of age at disease onset on disease activity and disability: Results from the Ontario Best Practices Research Initiative. Clin. Rheumatol..

[6] Corsetti M., Caenepeel P., Fischler B., Janssens J., Tack J. (2004). Impact of coexisting irritable bowel syndrome on symptoms and patho-physiological mechanisms in functional dyspepsia. Am. J. Gastroenterol..

[7] Wolfe F., Kong S.X., Watson D.J. (2000). Gastrointestinal symptoms and health related quality of life in patients with arthritis. J. Rheumatol..

[8] Chong V.H., Wang C.L. (2008). Higher prevalence of gastrointestinal symptoms among patients with rheumatic disorders. Singap. Med. J..

[9] Vitetta L.L., Coulson S., Schloss J., Allen S. (2012). Dietary recommendations for patients with rheumatoid arthritis: A review. Nutr. Diet. Suppl..

[10] Dos Santos A.T., Assunção A.A.Q., Foschetti D.A. (2016). Assessment of nutritional and biochemical status in patients with rheumatoid arthritis undergoing pharmacological treatment. A pilot study. Int. J. Clin. Exp. Med..

[11] Markaki A.G., Gkiouras K., Papakitsos C., Grammatikopoulou M.G., Papatsaraki A., Ioannou R., Tsagkari A., Papamitsou T., Bogdanos D.P. (2020). Disease Activity, Functional Ability and Nutritional Status in Patients with Rheumatoid Arthritis: An Observational Study in Greece. Mediterr. J. Rheumatol..

[12] Sokoll K.B., Helliwell P.S. (2001). Comparison of disability and quality of life in rheumatoid and psoriatic arthritis. J. Rheumatol..

[13] De Resende Guimarães M.F.B., Rodrigues C.E.M., Gomes K.W.P., Machado C.J., Brenol C.V., Krampe S.F., De Andrade N.P.B., Kakehasi A.M. (2019). High prevalence of obesity in rheumatoid arthritis patients: Association with disease activity, hypertension, dyslipidemia and diabetes, a multi-center study. Adv. Rheumatol..

[14] Świtała A., Wyszyńska J., Czerwińska K., Dereń K., Podgórska-Bednarz J., Guzik A. (2018). Association between body mass and physical activity with quality of life in patients with rheumatoid arthritis. Eur. J. Clin. Exp. Med..

[15] García-Poma A., Segami M.I., Mora C.S., Ugarte M.F., Terrazas H.N., Rhor E.A., García E., Ramos M.P., Alva M., Castañeda I. (2007). Obesity is independently associated with impaired quality of life in patients with rheumatoid arthritis. Clin. Rheumatol..

[16] Fukuda W., Yamazaki T., Akaogi T., Hayashi H., Kusakabe T., Tsubouchi Y., Kawahito Y., Inoue M., Yoshikawa T. (2005). Malnutrition and disease progression in patients with rheumatoid arthritis. Mod. Rheumatol..

[17] Gezer I.A., Balkarli A., Can B., Bagcaci S., Küçükşen S., Kucuk A., Balkarlı A. (2017). Pain, depression levels, fatigue, sleep quality, and quality of life in elderly patients with rheumatoid arthritis. Turk. J. Med Sci..

[18] Aziz E.F., Javed F., Pratap B., Musat D., Nader A., Pulimi S., Alivar C.L., Herzog E., Kukin M.L. (2011). Malnutrition as Assessed by Nutritional Risk Index is Associated with Worse Outcome in Patients Admitted with Acute Decompensated Heart Failure: An ACAP-HF Data Analysis. Hear. Int..

[19] Yamaya M., Usami O., Nakayama S., Tode N., Yamada A., Ito S., Omata F., Momma H., Funakubo M., Ichinose M. (2020). Malnutrition, Airflow Limitation and Severe Emphysema are Risks for Exacerbation of Chronic Obstructive Pulmonary Disease in Japanese Subjects: A Retrospective Single-Center Study. Int. J. Chronic Obstr. Pulm. Dis..

[20] Polański J., Jankowska-Polańska B., Uchmanowicz I., Chabowski M., Janczak D., Mazur G., Rosińczuk J. (2017). Malnutrition and Quality of Life in Patients with Non-Small-Cell Lung Cancer. Adv. Exp. Med. Biol..

[21] Aletaha D., Neogi T., Silman A.J., Funovits J., Felson D.T., Bingham C.O., Birnbaum N.S., Burmester G.R., Bykerk V.P., Cohen M.D. (2010). 2010 Rheumatoid arthritis classification criteria: An American College of Rheumatology/European League Against Rheumatism collaborative initiative. Arthritis Rheum..

[22] Folstein M.F., Folstein S.E., McHugh P.R. (1975). Mini-mental state. A practical method for grading the cognitive state of patients for the clinician. J. Psychiatry Res..

[23] Costa D., Severo M., Fraga S., Barros H. (2012). Mini-Cog and Mini-Mental State Examination: Agreement in a Cross-Sectional Study with an Elderly Sample. Dement. Geriatr. Cogn. Disord..

[24] Lawton M.P., Brody E.M. (1969). Assessment of Older People: Self-Maintaining and Instrumental Activities of Daily Living. Gerontologist.

[25] Sikkes S., Klerk E.S.M.D.L.-D., Pijnenburg Y., Scheltens P., Uitdehaag B. (2008). A systematic review of Instrumental Activities of Daily Living scales in dementia: Room for improvement. J. Neurol. Neurosurg. Psychiatry.

[26] Wołowicka L. Jakość Życia w Naukach Medycznych Tom I. https://www.umb.edu.pl/photo/pliki/Dziekanat-WNOZ/monografie/12-2017/jakosc_zycia_tom_i.pdf.

[27] Ilić I., Šipetić S., Grujičić J., Mačužić I.Ž., Kocić S., Ilić M. (2019). Psychometric Properties of the World Health Organization’s Quality of Life (WHOQOL-BREF) Questionnaire in Medical Students. Medicina.

[28] Rubenstein L.Z., Harker J.O., Salvà A., Guigoz Y., Vellas B. (2001). Screening for Undernutrition in Geriatric Practice: Developing the Short-Form Mini-Nutritional Assessment (MNA-SF). J. Gerontol. Ser. A Boil. Sci. Med Sci..

[29] Bleda M.J., Bolibar I., Parés R., Salvà A. (2002). Reliability of the mini nutritional assessment (MNA) in institutionalized elderly people. J. Nutr. Health Aging.

[30] Nowak A., Zep W., Straburzyńska-Lupa A., Romanowski W. (2012). Assessment of the nutritional value of food rations of women with rheumatoid arthritis. Reumatologia.

[31] Rasheed S., Woods R.T. (2014). An investigation into the association between nutritional status and quality of life in older people admitted to hospital. J. Hum. Nutr. Diet..

[32] Fukuda W., Omoto A., Ohta T., Majima S., Kimura T., Tanaka T., Kohno M., Kawahito Y. (2013). Low body mass index is associated with impaired quality of life in patients with rheumatoid arthritis. Int. J. Rheum. Dis..

[33] Kremers H.M., Nicola P.J., Crowson C.S., Ballman K.V., Gabriel S.E. (2004). Prognostic importance of low body mass index in relation to cardiovascular mortality in rheumatoid arthritis. Arthritis Rheum..

[34] Sierakowska M. (2017). Quality of life in chronic rheumatic diseases—Social, psychological and medical conditions and measurement methods. Forum Reumatol..

[35] Tada M., Yamada Y., Mandai K., Hidaka N. (2019). Correlation between frailty and disease activity in patients with rheumatoid arthritis: Data from the CHIKARA study. Geriatr. Gerontol. Int..

[36] Uchmanowicz I., Kuśnierz M., Wleklik M., Jankowska-Polańska B., Jaroch J., Łoboz-Grudzień K. (2017). Frailty syndrome and rehospitalizations in elderly heart failure patients. Aging Clin. Exp. Res..

[37] Marcora S.M., Chester K.R., Mittal G., Lemmey A.B., Maddison P.J. (2006). Randomized phase 2 trial of anti-tumor necrosis factor therapy for cachexia in patients with early rheumatoid arthritis. Am. J. Clin. Nutr..

[38] Salaffi F., Di Carlo M., Farah S., Di Donato E., Carotti M. (2019). Prevalence of frailty and its associated factors in patients with rheumatoid arthritis: A cross-sectional analysis. Clin. Rheumatol..

[39] Chen Y.-M., Chen L.-K., Lan J.-L., Chen D.-Y. (2009). Geriatric syndromes in elderly patients with rheumatoid arthritis. Rheumatology.

[40] Bąk E., Młynarska A., Marcisz C., Bobiński R., Sternal D., Młynarski R. (2020). Factors that affect the assessment of the quality of life of rheumatoid arthritis patients depending on the prevalence of frailty syndrome. Health Qual. Life Outcomes.

